# Commonly collected thermal performance data can inform species distributions in a data-limited invader

**DOI:** 10.1038/s41598-023-43128-4

**Published:** 2023-09-23

**Authors:** Natalie M. Claunch, Colin M. Goodman, Bryan M. Kluever, Narayani Barve, Robert P. Guralnick, Christina M. Romagosa

**Affiliations:** 1 USDA, APHIS, Wildlife Services, National Wildlife Research Center, Florida Field Station, Gainesville, FL USA; 2 Department of Wildlife Ecology and Conservation, University of Florida, Gainesville, FL USA; 3 Department of Biology, University of Florida, Gainesville, FL USA; 4 Department of Natural History, Florida Museum of Natural History, Gainesville, FL USA; 5 Department of Integrative Biology, University of South Florida, Tampa, FL USA

**Keywords:** Ecological modelling, Ecophysiology, Invasive species, Animal physiology

## Abstract

Predicting potential distributions of species in new areas is challenging. Physiological data can improve interpretation of predicted distributions and can be used in directed distribution models. Nonnative species provide useful case studies. Panther chameleons (*Furcifer pardalis*) are native to Madagascar and have established populations in Florida, USA, but standard correlative distribution modeling predicts no suitable habitat for *F. pardalis* there. We evaluated commonly collected thermal traits– thermal performance, tolerance, and preference—of *F. pardalis* and the acclimatization potential of these traits during exposure to naturally-occurring environmental conditions in North Central Florida. Though we observed temperature-dependent thermal performance, chameleons maintained similar thermal limits, performance, and preferences across seasons, despite long-term exposure to cool temperatures. Using the physiological data collected, we developed distribution models that varied in restriction: time-dependent exposure near and below critical thermal minima, predicted activity windows, and predicted performance thresholds. Our application of commonly collected physiological data improved interpretations on potential distributions of *F. pardalis*, compared with correlative distribution modeling approaches that predicted no suitable area in Florida. These straightforward approaches can be applied to other species with existing physiological data or after brief experiments on a limited number of individuals, as demonstrated here.

## Introduction

A key aim throughout many subfields of ecology is predicting where organisms occur. A common approach is to extract landscape and climate data from known occurrences for use in correlative distribution modeling^1,2^ but these require appropriate sampling of environmental preferences and have other limitations^3–5^. For example, correlative distribution modeling relies on extensive sampling of species presences and an assumption that these presences reflect the existing fundamental niche for the species^6,7^. Physiological data provide a basis for more mechanistic models and better inform prediction and forecasting of future distribution changes, especially for cryptic, rare, or introduced species^8–11^. While collecting physiological data is resource-intensive, doing so can provide information about flexibility of traits, revealing more about the overall fundamental niche (rather than the existing fundamental niche, a best-case outcome for correlative models;^6,7^) and potential distribution. It is not uncommon for species with restricted distributions to have far broader physiological tolerances, allowing them to establish beyond their known current distribution^5,12,13^.

A popular approach to integrating physiological data in predictive frameworks are mechanistic niche models, which utilize biophysics to derive physiological constraints and thresholds that can then be projected to the landscape-scale^14,15^. The demands of many mechanistic modeling frameworks require strong knowledge of species ecophysiology^16^ and thus are most useful for testing hypotheses in well-studied, model systems^7^ or in well-funded species where reduced time to accrue the data may not be a priority. While predictions would admittedly be more accurate with comprehensive knowledge of a species, in practice, answers are often required under short timelines with limited prior data and funding. To try and address these demands, we aimed to investigate the utility of models based on easy-to-collect, well known physiological thresholds derived from commonly collected data on physiological tolerances.

We used a species that is cryptic, rare, and introduced to explore the potential seasonal plasticity of physiological tolerance and performance and the utility of different physiologically derived metrics in predicting potential distributions. Of the many potential physiological constraints, we focused on thermal tolerance as it is especially important for determining distributions of ectotherms, which comprise the majority of species on earth^17^. We assessed thermal traits in the panther chameleon, *Furcifer pardalis*, which occurs in several introduced populations in Florida, USA with the earliest report from 2008^18^. Introduced populations of chameleons tend to go unreported and some have been “seeded” in areas for later collection due to their high value in the pet trade^19^. Lack of reporting obscures the knowledge of the current extent of introduced populations, presenting challenges for directing management and regulatory actions. Predicting the distribution of this species in particular is difficult for two main reasons. First, there is relatively little known on thermal limits of this taxon, owing to limited studies of their thermal biology and limited information from related species in the native range in Madagascar^20,21^. Second, the occurrence of populations in Florida at higher latitudes than the native range suggests a greater thermal tolerance than predicted based on its native range in Madagascar or invasive range of Réunion^22^. Panther chameleons have a fast life-history strategy, typically maturing within 14 months^23^, allowing for the possibility of rapid adaptation of introduced populations to local thermal regimes. Thus, we opted to test the thermal traits of chameleons from a population from the highest known latitude^24^, to account for potential extremes in thermal tolerance.

We assessed thermal tolerance, thermal performance, and thermal preference of *F. pardalis* exposed to seasonal fluctuations in climate. We hypothesized that chameleons would show a shift in thermal traits associated with seasonal exposure; in particular, we predicted that thermal preference may decrease and cold tolerance and performance at low temperatures would improve following exposure to winter conditions. We then used these trait data and fine-grain, daily temperature data to predict suitable winter habitats across Florida. We also constructed correlative niche models using native range occurrence data, which is often the only option for predicting suitable habitats in data-limited non-native species when physiological data are not available. We then evaluated which models and which thermal traits may be most useful for such predictions. We predicted that the correlative niche model would under-perform compared to models derived from physiological trait data, because of the apparent niche-shift in *F. pardalis* in Florida. Of the models integrating physiological trait data, we predicted that models integrating critical thermal minima would be most restrictive, followed by those integrating preference and performance.

## Results

### Thermal limits

**Figure 1:**
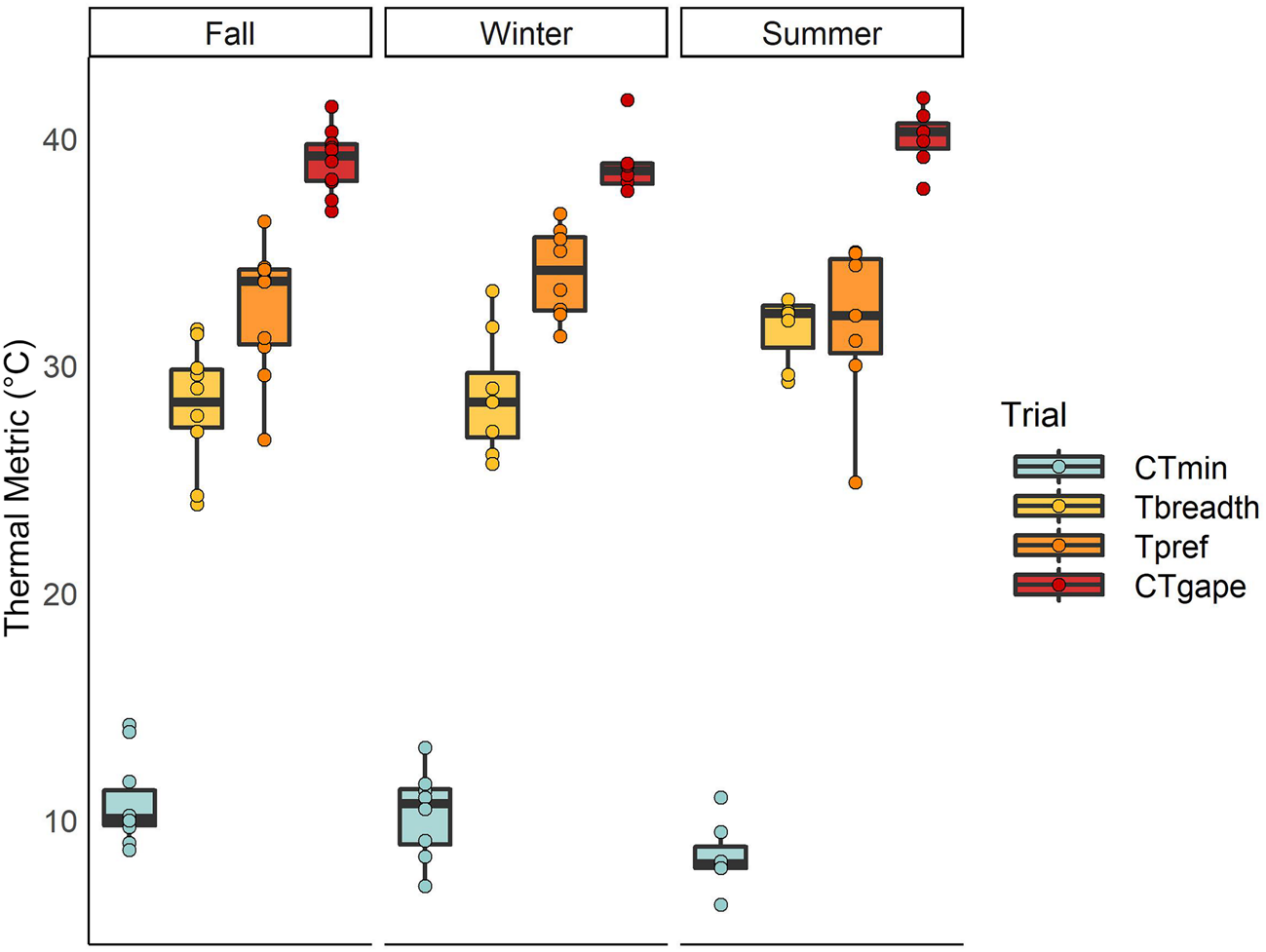
Thermal metrics and associated standard errors from chameleons collected from the northernmost established population of *Furcifer pardalis* in Florida, USA after exposure to seasonal fluctuations in temperature at USDA Wildlife Services National Wildlife Research Center in Gainesville, Florida, from October 2020 to August 2021. *CT*_min_ refers to the critical thermal minimum, the temperature at loss of righting response; *CT*_gape_ refers to the gaping threshold, the temperature at which chameleons gape to thermoregulate; *T*_breadth_ refers to the difference between the *CT*_gape_ and *CT*_min_; *T*_pref_ refers the preferred body temperature, as the average body temperature selected in a thermal gradient.

Body size of chameleons was 13.6 + /− 2.5 cm snout to vent length (SVL) and 70.7 + /− 33.5 g. *CT*_min_ averaged 9.9 °C + /− 2.0 (SD), with an average rate of chameleon temperature change of -0.6 °C + /− 0.3 (SD) per minute. There was an effect of season on critical thermal minimum (*CT*_min_; F_2__,__9_ = 4.31, *p* = 0.05), though Tukey post-hoc tests did not reveal significant differences among seasons (all *p* > 0.1; Fig. 1). *CT*_min_ did not appear to be affected by which trial was experienced first (F_1__,__9_ = 3.85, *p* = 0.08). We did not find an interaction between chameleon temperature rate change and mass (F_1__,__9_ = 0.80 *p* = 0.39), and neither mass nor temperature rate change alone influenced *CT*_min_ (F_1__,__9_ = 3.06, *p* = 0.11; F_1_, _9_ = 0.52, *p* = 0.49, respectively).

*CT*_gape_ averaged 39.2 °C + /− 1.4 (SD), with an average rate of chameleon temperature change of 0.6 °C + /− 0.2 (SD) per minute. We did not find an effect of season on the high temperature at which gaping occurred (*CT*_gape_; F_2__,__9_ = 2.18, *p* = 0.17; Fig. 1). *CT*_gape_ was not affected by which trial was experienced first (F_1__,__9_ = 0.08, *p* = 0.79). We did not observe an interaction between chameleon temperature rate change and mass (F_1__,__9_ = 0.56, *p* = 0.48), nor did we observe an effect of mass (F_1__,__9_ = 0.35, *p* = 0.57). Rate of chameleon temperature change was negatively correlated to *CT*_gape_, such that an increase of heating rate by 1 °C per minute led to lower *CT*_gape_ by 6.6 °C (F_1__,__9_ = 6.18, *p* = 0.04).

Average *T*_breadth_ was 29.3 °C + /− 2.7 (SD). There was an effect of season on thermal breadth (*T*_breadth_; F_2__,__11_ = 5.69, *p* = 0.02), although Tukey post-hoc tests did not distinguish significant differences in *T*_breadth_ among the seasons (all *p* > 0.1; Fig. 1). *T*_breadth_ was not affected by which trial was experienced first (F_1__,__11_ = 2.84, *p* = 0.12), nor was it influenced by mass (F_1__,__11_ = 1.81, *p* = 0.21).

### Thermal preference

The maximum body temperature reached during a preference trial was 40.6 °C and the minimum was 21.9 °C. Average preferred body temperature was 32.8 °C + /− 2.9 and did not differ by season (F_2__,__12_ = 1.23, *p* = 0.33; Fig. 1), and was not affected by mass (F_1__,__12_ = 0.011, *p* = 0.919). The body temperature exhibited most often by each chameleon (mode) was 34.2 °C + /− 4.7, and also did not differ by season (F_2__,__12_ = 2.78, *p* = 0.10) and was not affected by mass (F_1__,__12_ = 0.32, *p* = 0.59). The standard deviation of each chameleon’s body temperature during a trial was 2.9 °C and also did not differ by season (F_2__,__12_ = 2.78, *p* = 0.10) and was not affected by mass (F_1__,__12_ = 0.32, *p* = 0.59).

### Thermal performance

**Figure 2:**
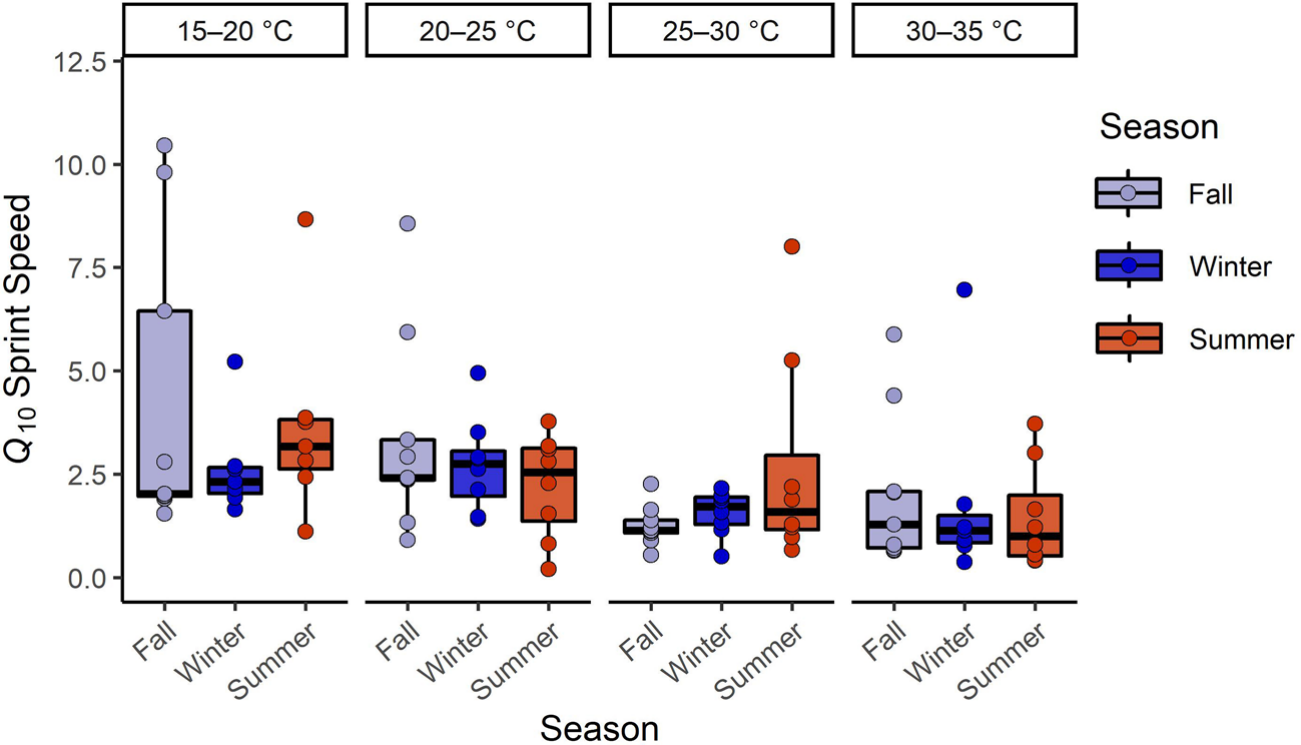
*Q*_10_ values calculated from maximal sprint speed for four temperature intervals tested in individuals from the northernmost established population of *Furcifer pardalis* in Florida, USA after exposure to seasonal fluctuations in temperature at USDA Wildlife Services National Wildlife Research Center in Gainesville, Florida, from October 2020 to August 2021. These values represent the magnitude of increase in a rate with a 10 °C increase in temperature, such that a value of two represents a doubling the rate per 10 °C increase, values of one represent thermal independence, and values of 0.5 represent a halving of the rate per 10 °C increase.

Thermal sensitivity of sprint performance (*Q*_10_) differed significantly among temperature intervals (F_3__,__80_ = 4.71, *p* = 0.005). Tukey post hoc tests revealed that *Q*_10_ values for the 15–20 °C interval (x̅ = 5.08) differed significantly from the 25–30 °C interval (x̅ = 1.86, *p* = 0.01) and from the 30–35 °C interval (x̅ = 4.50, *p* = 0.008), but not the 20–25 °C interval (x̅ = 2.78, *p* = 0.43). *Q*_10_ values were not affected by SVL (F_1__,__80_ = 0.008, *p* = 0.93). Additionally, season had no impact on thermal sensitivity of sprint performance (F_2__,__80_ = 0.052, *p* = 0.95; Fig. 2).

### Correlative distribution model

The top model (Supplementary Fig. S4) predicted no suitable habitat for *F. pardalis* in Florida, despite established populations existing there. The top model had a regularization multiplier of 2 and used feature classes of linear, quadratic, hinge, product, and threshold, with AICc = 3288.793 and AUC of 0.916 (see data repository for details). The final set of layers and their percent contribution consisted of: temperature annual range (bio7, 82.32%), precipitation of wettest month (bio13, 12.53%), maximum temperature of the warmest month (bio5, 2.04%), precipitation of the warmest quarter (bio18, 2.01%), and isothermality (bio3, 1.10%).

### Integrative distribution models

**Figure 3:**
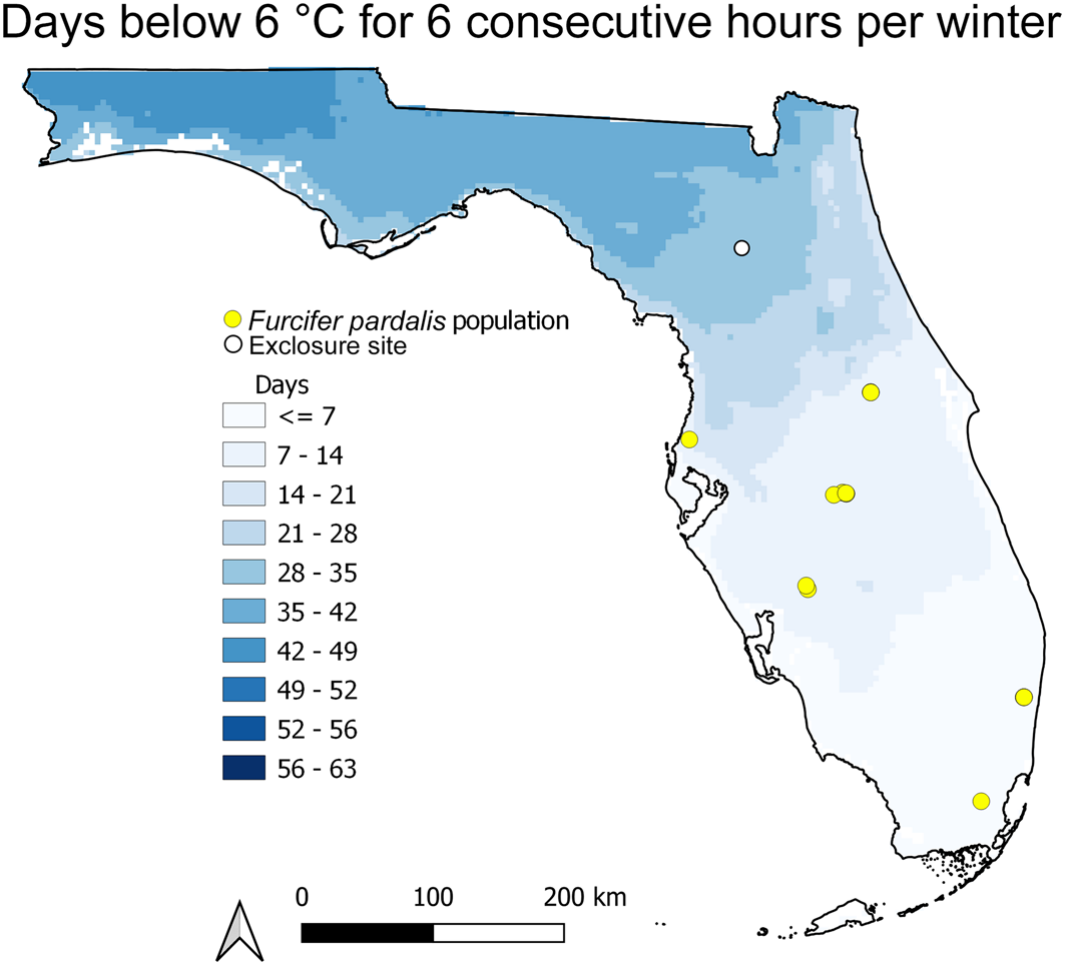
Average number of days during each winter (Dec 15–Feb 15) in Florida, USA, where temperature fell below the lowest critical thermal minimum of *Furcifer pardalis* for 6 or more hours, from 2001 to 2021. Yellow points indicate locations where *F. pardalis* populations have established. White point indicates the location of the experimental exclosure at USDA Wildlife Services National Wildlife Research Center in Gainesville, Florida.

**Figure 4:**
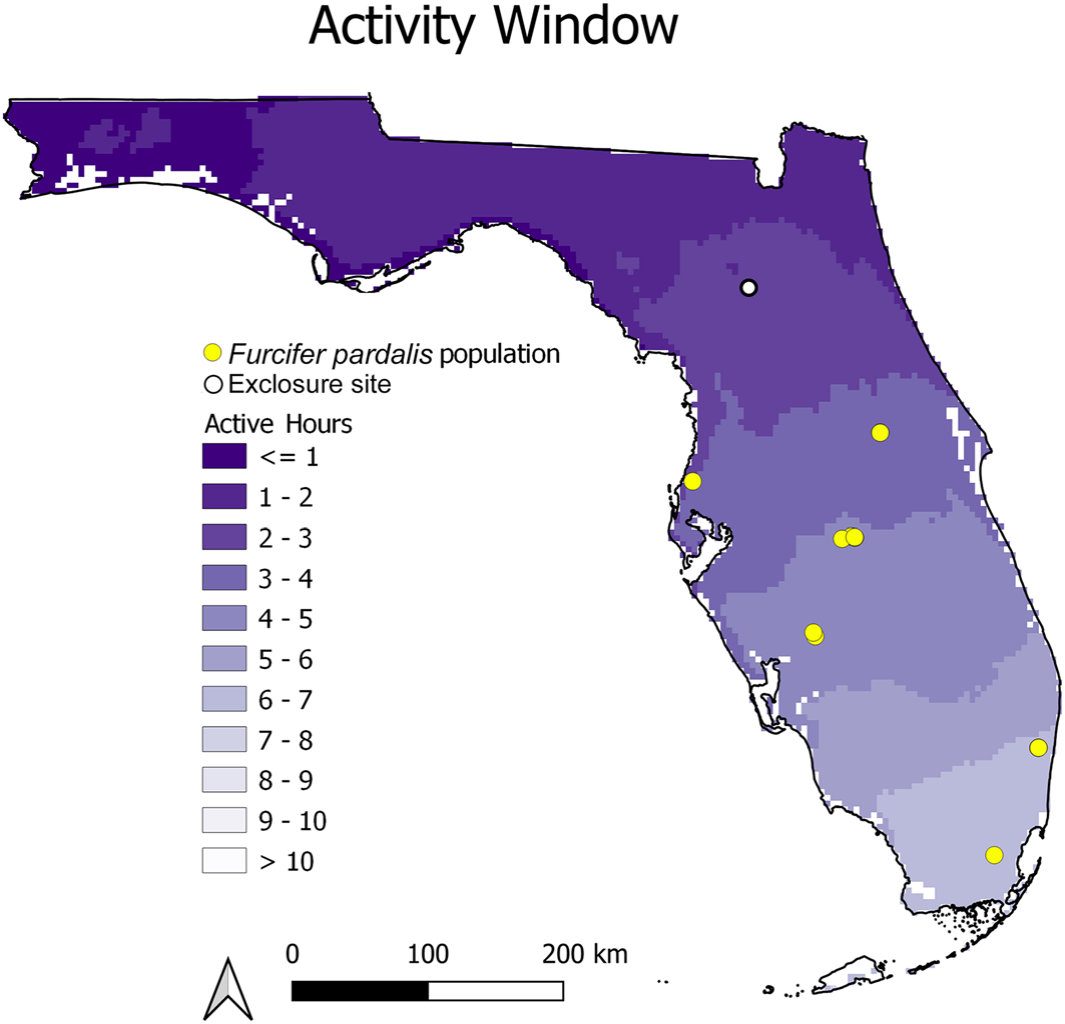
The average number of potential daylight hours *Furcifer pardalis* may be active in Florida, USA during the winter (Dec 15–Feb 15) from 2001 to 2021, based on thermal preference data for the northernmost population of this species in Florida. There are 11 possible total hours of daylight during this winter period. Yellow points indicate locations where *F. pardalis* populations have established. White point indicates the location of the experimental exclosure at USDA Wildlife Services National Wildlife Research Center in Gainesville, Florida.

**Figure 5:**
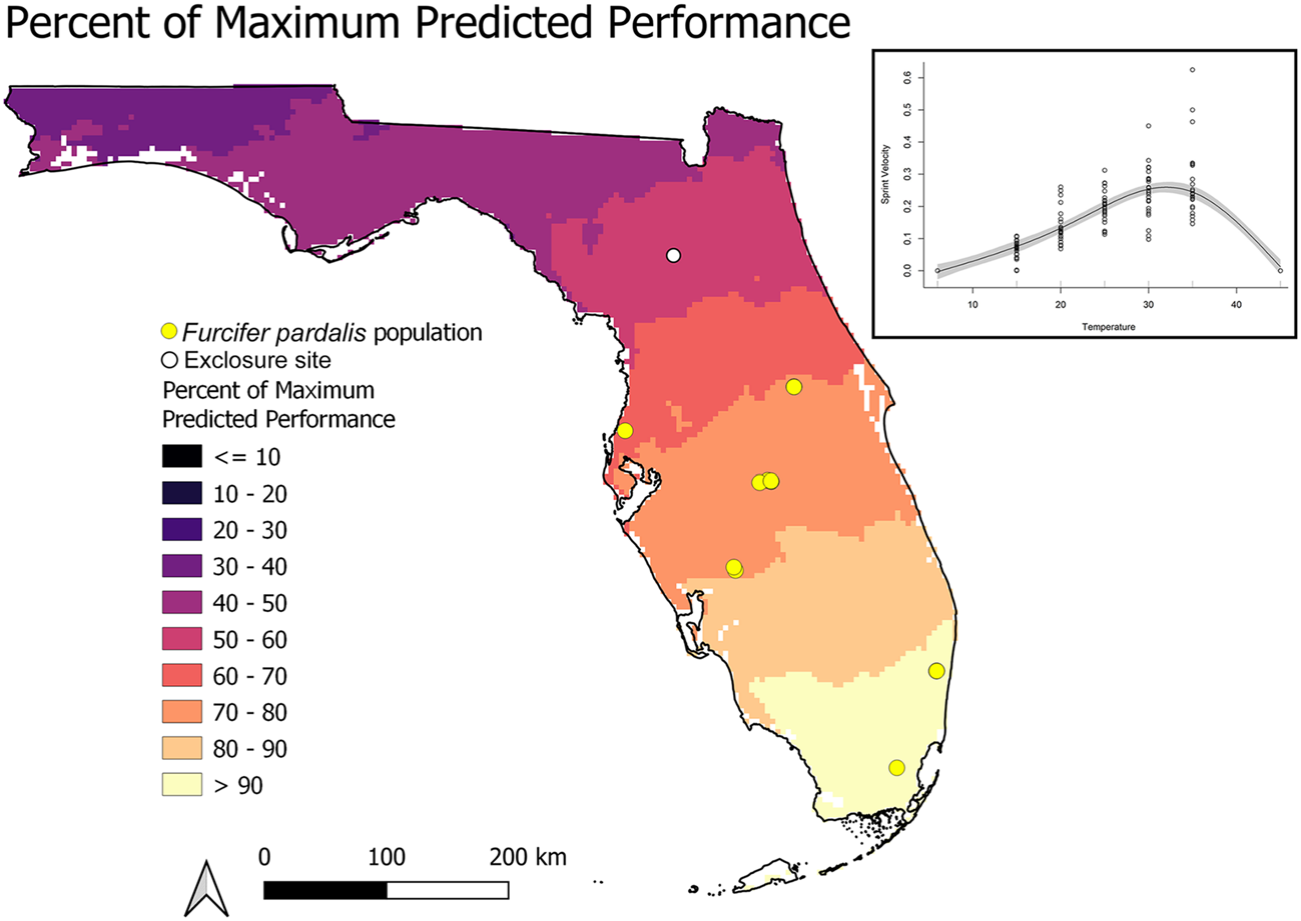
The predicted performance of *Furcifer pardalis* in Florida, USA, expressed as a percentage of the maximum predicted performance value from a generalized additive model of thermal performance data from the northernmost population of this species in Florida (inset). Yellow points indicate locations where *F. pardalis* populations have established. White point indicates the location of the experimental exclosure at USDA Wildlife Services National Wildlife Research Center in Gainesville, Florida.

Summary data extracted from model projections at *F. pardalis* presences (*N* = 9 distinct presences within the 1 km resolution of PRISM data) are presented in Table 1. *F. pardalis* populations have established in areas where the winter temperatures fell below the lowest critical thermal minimum for 6 consecutive hours an average of fewer than 10 days per season (Fig. 3, Table 1). At the other two thresholds, populations have established in areas experiencing the temperature threshold for under one month each winter (9 °C, near average *CT*_min_) and 5 days per season (3 °C, below *CT*_min_ and exposed temperatures; Table 1; see Supplementary Fig. S5 online). The average activity window estimated for areas *F. pardalis* have established falls between 2.97 and 6.28 h of 11 h of available daylight per day (Table 1), and this window decreases with increasing latitude (Fig. 4). At the highest latitude populations, there is an average of 3–4 h per day through the winter period where ambient air temperatures are within the preferred temperatures of *F. pardalis* (Fig. 4). The entire state of Florida falls within 80% of the average predicted performance for *F. pardalis* during the winter period (Fig. 5), and the predicted performance at areas *F. pardalis* have established exists in a relatively narrow range of 44.5–63.2% (Table 1). The normalized activity window and predicted performance were relatively similar in their predictions; however, the activity window was slightly more conservative in its output, placing established populations in a threshold 10% more restrictive than the performance model (see Supplementary Fig. S6 online).

**Table 1:** Summary of values associated with presence localities (*N* = 9) of *Furcifer pardalis* derived from experimental data on *F. pardalis* and climate data from winter 2001 to 2021. Days below a temperature threshold represent the average number of days per winter season that fell below the associated temperature threshold for a consecutive 6 h or more. Active Hours represents the average number of estimated active daylight hours per day across all winters. Percent of Daylight Active represents the percentage of hours during daylight that fall within the activity window for *F. pardalis*. Performance represents the average predicted performance represented as a % of maximum across all winters.

Metric	Days per winter below 3 °C	Days per winter below 6 °C	Days per winter below 9 °C	Active hours	Percent of daylight active	Performance
Min	0.15	1.7	5.45	2.97	26.97%	44.54%
Max	4.8	12.9	28	6.28	57.13%	63.18%
Average	3.06	9.03	20.77	4.55	41.36%	53.61%

## Discussion

We conducted the first tests of seasonal acclimation of thermal traits in *Furcifer pardalis*, and found that *CT*_min_, *CT*_max_, *T*_pref_, and performance were not influenced by season. We anticipated seasonal acclimatization in thermal traits after exposure to low temperatures. For example, increased cold tolerance is reported after acclimation to cool temperatures in other arboreal lizards^12,25^; increases in thermal preference follow increases in ambient temperatures in newts^26^; and introduced geckos prefer lower temperatures in winter^27^. Our general lack of observed seasonal acclimation may be partly influenced by experimental choice; namely exposure of chameleons to natural temperature fluctuations rather than constant temperature treatments in each season, combined with natural variation in acclimation ability among individuals^28^, but see^29^. In other tropical species, extended constant exposure to low temperatures may be necessary to observe acclimation of thermal traits e.g.^30^. The influence of seasonal acclimatization on thermal traits varies widely^31^. Some species display acclimation of certain thermal traits, but not others. For example, introduced curly-tailed lizards show seasonal acclimation in *CT*_max_ but not *CT*_min_^32^, and in the frog *Pleurodema thaul* thermal performance and preference were not influenced by acclimation temperature, but thermal tolerance was^33^. Other species may show inter-population variation in acclimation. In *A. cristatellus*, one population demonstrates ability to acclimate to cold temperatures, but another population of the same species does not^12^, while in the armadillo girdled lizard (*Ouroborus cataphractus*), two populations maintain similar thermal preference despite seasonal change^34^. Discrepancies and patterns in acclimation of thermal traits are well documented elsewhere e.g.^31^, and the underlying mechanisms for these deserve further study.

In the case of *F. pardalis,* a potential explanation for lack of observed acclimatization is that the study population may have adapted to local thermal conditions to express conserved thermal traits year-round. The fact that the individuals we tested are from the highest latitude documented for the species^18^, that *F. pardalis* have relatively fast generation times^23^, and that this species does not typically burrow or seek shelter to buffer from cold temperatures (Claunch *pers obsv*) lends credence to the possibility of local adaptation. Adaptation without acclimation is not unprecedented. Another tree-dwelling lizard, *A. cristatellus*, demonstrates differences in *CT*_min_ among introduced and native populations, without associated acclimation ability in one of the introduced populations^25^. Local adaptation of *CT*_max_^35^; *CT*_min_^36,37^ and thermal preference^27^ are documented among various lizard populations. It is important to note that our study is not equipped to test whether adaptation or acclimatization had occurred in the *F. pardalis* population prior to our testing. Unfortunately, our attempts to include chameleons from lower-latitude populations in a common-garden style comparison were thwarted due to collection-depletion and lack of public access at documented sites. The native-range origin of our study animals is also not known with certainty—coloration suggests they may be hybrids from multiple geographic origins^18^. Our preferred body temperature result is slightly higher than a previous study on *F. pardalis*—where Ferguson et al.^20^ report preferred temperatures from three individuals as 31 °C, we report slightly higher preferred temperatures approaching 33 °C. While gaping thresholds are not reported, Ferguson et al.^20^ report panting observed above 36 °C; this may represent the lowest observed panting threshold, as gaping typically precedes panting and we report gaping at temperatures averaging around 40 °C. Expanded sampling of multiple populations will be necessary to determine whether this population is representative of inherent thermal plasticity in *F. pardalis* or represents improved cold tolerance at the extremes of thermal plasticity in the species.

As anticipated, the correlative niche modeling approach, which did not integrate physiological data, under-predicted suitable habitat in the non-native range in Florida. This can largely be attributed to using native occurrences to model the invaded range. We chose this approach because it is the best practice approach in species distribution modeling of invasive species. Modeling invaded ranges using occurrences from within that range is problematic because of the lack of presumed equilibrium with the environment and often sparse occurrence data^4^. These issues especially limit the ability to effectively forecast distributions of nonnative species under climate changes^7,38^. While it is possible to combine native and nonnative populations into a joint model of *F. pardalis* distribution, the data density remains weighted strongly towards the native range and the end result is a model tuning exercise to determine how to balance omission and commission errors, without a clear external means to assess optimal model quality. Here, our native-range model is not useful for informing about potential distributions of *F. pardalis* in Florida, but may instead provide important baseline niche information, which can be used to compare the magnitude of potential niche shift.

Models directly integrating physiological information may be the most effective way to improve predictions of potential distribution of invaders^8,39,40^. Of the physiological traits we tested, cold tolerance is likely the most limiting factor for *F. pardalis* range expansion in Florida, given what we know about its native abiotic niche. Our lack of observed seasonal differences among thermal traits justify the use of average trait values, which simplifies modeling approaches and interpretation. Namely, our model predictions would remain largely unchanged if we had only used thermal trait data derived from a single sampling effort. Additionally, because we tested individuals from a population that is of higher latitude than its native range, data from this population may give a decent approximation of the limits of thermal flexibility of the species. At the very least, we demonstrate that physiological tolerance information even on a limited number of individuals can provide a better estimate of occurrence than comparatively abundant occurrence data from the native range. By integrating physiological data from populations at the edge of their niche into niche modeling frameworks, we can gain a more accurate picture of the niche limits of this species than is possible with occurrence data alone.

Our use of consecutive hours at each minimum temperature threshold is a conservative method to apply physiological data to understanding chameleon occurrence. Ectotherms can often survive brief exposure to temperatures below their *CT*_min_, while longer exposure can lead to death, e.g.^41^. By considering length of exposure to cold thresholds, we may gain a more ecologically relevant insight into distribution limits and elucidate where population-limiting temperature thresholds occur. As expected, as temperature thresholds decrease, the average number of Florida-wide occurrences of each threshold also decreases. In areas where chameleons have established, there are fewer instances of potential exposure to cool temperatures. Chameleons are potentially exposed to 4 total weeks where temperatures are 1 °C below their average *CT*_min_ for at least 6 consecutive hours; whereas they potentially experience 2 weeks of temperatures at the lowest measured *CT*_min_, and less than one week at the 3 °C threshold (Fig. 3; see Supplementary Fig S5 online). Existing populations showed less variation in 6-h exposures to the lowest two temperature thresholds than near the average *CT*_min_. That the near-average *CT*_min_ threshold was not as useful as the lowest and below-*CT*_min_ thresholds suggests that the more extreme cold tolerance values more closely represent population-limiting temperature exposures, especially when considering the temperature values used in thresholds were derived from brief exposures to determine *CT*_min_.

The use of average critical thermal limit thresholds in distribution models has been criticized when applied as a filter of single-value temperature occurrences in a landscape (i.e., when not accounting for exposure time;^42^), but also because *CT*_min_ is often several degrees above the lethal minimum temperature^16^. In some cases species may be immobilized by cold but are able to survive long bouts of cold exposure^43^. Lower lethal temperatures are unknown in *F. pardalis*. Ethical concerns aside, lethal temperature values may be too restrictive an approach to predicting suitable habitat. For example, a chameleon experiencing a chill coma (a temperature at or below *CT*_min_) may not die directly from low temperatures but the restricted foraging efforts, increased vulnerability to predators, and dampened immune function at low temperatures may manifest as population-level effects that prevent sustained survival of populations at higher-than-lethal temperature thresholds. The range in intra-species thermal tolerance can serve as a starting point for developing informative temperature thresholds on activity restriction in a species. A more holistic approach integrates chill coma temperatures or *CT*_min_ as well as higher temperatures where activity and performance are reduced but may still limit population survival.

Activity window and thermal performance thresholds provide perspective on potential behavioral limitations using ambient temperature data. Calculation of activity windows from thermal trait data is not new; there are many frameworks used to estimate activity windows and activity budgets, ranging from models requiring many data inputs, (e.g.^44–46^), to simpler threshold-based inputs (e.g.^47^). Our approach differs slightly from others in the calculation of hourly trait values, and in our choice of thermal preference or selected body temperature data as the basis for an activity window. The range of body temperatures we recorded (22–40 °C) in the thermal preference trial encompasses the range of field body temperatures reported from *F. pardalis* in Madagascar exhibiting normal activity (24–36 °C^20^). This demonstrates that our thermal preference data accurately reflect an activity window for the species. Our normalized data comparisons confirm that our activity window data derived from thermal preference are more restrictive than performance data. This is expected, as our activity window is constrained to temperature values chameleons chose, while the performance data clearly demonstrate that chameleons are capable of activity at higher and lower temperatures when induced to move.

The activity window and thermal performance thresholds represent different constraints and should be interpreted in different ways. The activity window, derived from thermal preference data, more likely demonstrates propensity to forage or explore, whereas temperatures outside this window are more likely directed towards seeking thermal refugia such as spending time basking to raise temperatures to levels where foraging can occur. The performance threshold more likely represents the ability to respond when extremely motivated to move, such as during pursuit by a predator. However, performance data may not be as useful a threshold in *F. pardalis* as it is for some other ectotherms. First, no area in Florida fell below 20% of the maximum predicted performance for this species, and predicted performance was around 50% at established populations, so variation in this trait was not particularly informative when applied to Florida winters. Second, sprint data may not correlate to success of escape from predators, as chameleons are fairly slow reptiles even at their best performance. Thermal preference data thus may be more biologically relevant, because if chameleons choose not to forage outside of their preferred temperatures they may not meet energy requirements to survive the winter. It is important to note these models do not account for potential radiative heating of basking chameleons to combat low ambient temperatures^48^, and conversely do not account for convective or conductive cooling during wind or rainfall. Indeed, the combination of fine-scale behavior data with radiation, windspeed, and precipitation data may improve the resolution of predictions and has been proposed in more complex frameworks that also estimate activity budgets (e.g.^44,45^). We chose to use only ambient temperature data as this is most commonly available^49^ and may be the only climate data consistently available in regions of the world where ectotherm biodiversity is highest^50–53^. We argue the value of our simple framework is that it can be applied in data-limited contexts, especially as global, fine-scale daily temperature data are now available worldwide^49^.

## Conclusions

We have shown that easy-to-collect physiological thermal trait and ambient temperature data can be used to accurately predict distributions of ectothermic organisms, in absence of native-range occurrence data. The thresholding approaches applied herein are relatively simple to execute, and limitations lie with computing power (which is typically not prohibitive), depending on the resolution and extent of predicted areas. While our case study focused on a non-native animal in an introduced range, the techniques herein are applicable beyond predicting distributions of invasive species. While mechanistic modeling approaches can have great utility for conservation with adequate inputs^54^, the majority of terrestrial ectothermic diversity and in turn the majority of data-deficient and at-risk ectotherms with limited occurrence data are unlikely to be candidates for more sophisticated mechanistic modeling approaches^7^. However, daily global temperature data are becoming available^49^, which creates opportunities for modeling approaches such as ours, which apply commonly available thermal trait data. These methods can thus be applied to improve understanding of distributions in data-deficient, rare, or threatened species using physiological data derived from a limited number of individuals. The methods could also be applied to simulate future distributions using predicted daily climate data. Finally, we provide predictions of potential occurrences of *F. pardalis* in Florida that may help focus management surveillance efforts. The limitations and accuracy of our approaches for this particular species will become clearer as additional populations are located.

## Methods

### Animal collection and housing

Ten chameleons were captured from an established non-native population in Central Florida from October 2019-February 2020 (*N* = 7 males, 2 females) and October 2020 (*N* = 1 male). Adult panther chameleons were housed individually in an experimental mesh exclosure at the US Department of Agriculture (USDA), Wildlife Services, National Wildlife Research Center, Florida Field Station, located approximately 115 km north of the collection site in Gainesville, Florida from September 30, 2020, to July 28, 2021. Conflicts with residents at the site of the established population prevented collection of more individuals^24^. Nine of the chameleons were housed by the authors prior to accessioning at USDA; the tenth was accessioned directly from the wild population to the exclosure in October 2020 (detailed in^55^). Animals were housed individually in screen enclosures with natural vegetation and exposure to natural sunlight and weather patterns in the greater exclosure (see Supplementary Fig. S1 online). An automated misting system provided dripping water for drinking four times daily. Chameleons were fed every other day with crickets dusted with calcium without D3 (Rep-Cal, Rep-Cal Research Labs, Los Gatos, California, USA) at every feeding, except when replaced with a multivitamin (Reptivite, Zoo Med, San Luis Obispo, California, USA) dusting once every other week. Chameleons received visual wellness checks once daily. Chameleons were exposed to natural thermal regimes, including low nighttime temperatures (12 °C) in winter (Fig. 1). On nights forecast below 12 °C, 250 W heat emitters were turned on over the cages and tarps placed to cover the sides and roof (see Supplementary Fig. S1 online). On nights forecast below 7 °C, chameleons were brought indoors (65–70 °C) and held in individual cloth bags. Thermal data loggers (iButton DS1922L, resolution 0.06 °C, accuracy 0.5 °C, Maxim Integrated, San Jose, California, USA) were placed within screen enclosures to collect data on variation in ambient temperatures (see Supplementary Fig. S2 online). All protocols were approved by USDA QA-3214 (Study Director, Bryan Kluever) and University of Florida IACUC 201,910,938.

### Assessing thermal traits

Thermal limit thresholds, preferences, and performance of chameleons were evaluated in three seasons: November 2020 (Fall), February 2021 (Winter), and July 2021 (Summer; see Supplementary Fig. S2 online). At each season, we collected mass using spring scales (Pesola Präzisionswaagen AG Schindellegi, Switzerland 0100 and 40,300 1 g and 2 g resolution, respectively) and SVL using a measuring tape (1 mm resolution). Animals were not evaluated in Spring due to outbreak of fungal infection (see^55^). All animals were de-accessioned from the study as of 1 August 2021. Chameleons were assessed for critical thermal limit thresholds before preference or performance trails were conducted. We conducted linear mixed models using the lme function in package nlme^56^ in R^57^. The alpha level for all statistical tests was set at 0.05.

### Critical thermal limit thresholds

Due to the proximity of critical thermal maximum to lethal maximum in some animals^58,59^ and the limited number of animals available for testing, we opted to measure the body temperature at which gaping occurred as a heat stress response (*CT*_gape_) as our upper thermal threshold. We used the body temperature at loss of righting response (*CT*_min_) as our lower temperature threshold.

At each season, individuals were randomly assigned to be tested first for either *CT*_gape_ or *CT*_min_. After full recovery from each threshold, chameleons were returned to their cages and were tested the following day for the remaining threshold. Animals were acclimated to room temperature (26 °C) for at least one hour prior to conducting thermal limit threshold measurements. A thermal probe was inserted into the cloaca and secured with medical tape to allow continuous recording of animal body temperature every 10 s throughout the trial. For *CT*_min_, animals were placed into a cooling incubator with windows (Benchmark Scientific, Sayreville, New Jersey, USA) set to 6 °C. When body temperatures were below 15 °C, we placed chameleons on their side with a gloved hand to test for righting response every 1.5 min and every 1 °C decrease in body temperature, whichever occurred first. When an animal was unable to right itself for 10 s after being flipped onto its side, we considered this *CT*_min_, recorded the time, and removed the animal from the incubator to recover. For *CT*_gape_, animals were placed into a heated incubator with windows (Labnet International Inc, Woodbridge, New Jersey, USA) set at 45 °C. We considered the gaping threshold reached when an animal held its mouth open for at least 5 s. We then recorded the time and removed the animal from the incubator to recover. All animals recovered from thermal limit threshold testing without incident. We extracted body temperatures for each threshold at the time each threshold was reached from the thermal logger data. We calculated the thermal breadth (*T*_breadth_) for each season for each individual by subtracting the *CT*_min_ from the *CT*_gape_ value.

We conducted linear mixed models with gaussian error distribution with fixed response variables *CT*_min_, *CT*_gape_ and *T*_breadth_. Because rates of temperature change can influence thermal limit thresholds^12,42^ we calculated the average rate of body temperature change per minute during the trial and included this as a covariate in *CT*_min_ and *CT*_gape_ analyses. In the thermal threshold models we included the following variables: season, which trial was experienced first (i.e. *CT*_min_ or *CT*_gape_ trial), and the interaction between rate of body temperature change and mass. In the *T*_breadth_ model we included season, which trial was experienced first, and mass as covariates. To account for repeated measures, animal ID was included as a random intercept effect in all models. Animals that were moribund or in poor body condition were excluded from analyses (*N* = 1 female in winter, *N* = 1 male in summer; see Supplementary Fig. S2 online). Where a factor variable (e.g., season) indicated significance at a threshold of alpha = 0.05, we conducted a Tukey post-hoc test using package emmeans^60^ in an attempt to discriminate differences among factor levels.

### Thermal preference

To assess thermal preference, a thermal gradient was created in a 1.25 m by 2 m arena divided in half to create two lanes. To facilitate use by chameleons, two wooden dowels were inserted into the center of each lane. At one end, two 250 W and two 150 W ceramic heat emitters were arranged to provide a hot environment to 51 °C ambient temperature. The other end was surrounded by ice packs and a bucket of salted ice with two small electronic fans (Shenzhen Glovion Technology Co., Shenzhen, China) to maintain cool air flow to 18 °C ambient temperature (see Supplementary Fig. S3 online). A curtain was drawn to prevent chameleons from reacting to researcher presence. Chameleons were monitored occasionally from behind the curtain to ensure they remained on the dowels. Chameleons had a thermocouple (0.076 mm diameter, Item 5SRTC-TT-K-40–72, Omega Engineering) inserted into the cloaca and secured with medical tape, attached by a 1.8 m lead to a datalogger (Item# OM-HL-EH-TC, Omega Engineering, resolution 0.1 °C, accuracy 0.8 °C) set to collect temperatures every 10 s for 65 min. The length of thermocouple leads and their small size and weight allowed for unrestrained movement throughout the area. Chameleons were initially placed in the middle of gradient. The first 5 min of data after animals were introduced to the arena were discarded to account for an acclimation period to the arena and after thermocouple insertion.

We extracted the average, mode, and standard deviation in body temperature from each chameleon’s thermal preference trial thermal logger data for analysis. Two chameleons’ thermocouples fell out within 15 min of the end of the trial (Fall season), and two chameleons exited the gradient mid-trial at which point they were replaced into the gradient and the portion of data where the chameleon was outside the gradient plus one minute after being replaced was discarded (Fall season). We used the remaining within-gradient trial data for analysis for these cases.

After confirming normality of data, we conducted a linear mixed model on the response variables of average, mode, and standard deviation of body temperature during the thermal preference trials. To account for repeated measures, we included chameleon ID as a random effect. We included mass and season as covariates. All chameleons walked to the hot end of the gradient after initial placement, thus starting choice was not included in analysis.

### Thermal performance

To assess thermal performance we tested chameleon sprint speed at five different body temperatures. Chameleons were randomly assigned to be tested at either “warm” (30 and 35 °C) or “cool” (15, 20, and 25 °C) temperatures per day. To achieve the assigned body temperature, chameleons were placed into either a cooling or heating incubator (described above in Critical Thermal Limit Thresholds) and continually monitored until the target temperature was reached, indicated by thermocouple in the cloaca. After reaching the target temperature, thermocouples were removed, and we placed chameleons at one end of a rubber mat divided into six, 0.25 m segments, and encouraged them to sprint across the mat by simultaneously tapping their tail gently with a gloved hand and luring with a bamboo branch ahead of the chameleon as it moved to the other side. During this, an observer used a stopwatch to record the time for the tip of the chameleon’s snout to pass each 0.25 m segment (segment time). Upon completing the entire length of the mat (1.5 m), we immediately repeated the process at the starting point of the mat; thus every trial consisted of two “laps”. Chameleons were allowed a minimum of 30 min rest between trials and chameleons completed two trials at each temperature. If the chameleon refused to move or was uncooperative (i.e., clearly performed submaximally), we denoted trials as being unsuccessful and excluded these trials from further analyses. We calculated the velocity of each segment by dividing the distance by segment time. For each acclimation temperature and chameleon, we retained only the maximal sprint speed over a 0.25-m segment for further analysis.

To compare the thermal sensitivity of sprint performance among seasons, we calculated *Q*_10_ values for each temperature interval. These values represent the magnitude of increase in a rate with a 10 °C increase in temperature, such that a value of two represents a doubling the rate per 10 °C increase, values of one represent thermal independence, and values of 0.5 represent a halving of the rate per 10 °C increase. *Q*_10_ values were calculated using the following equation^61^:

1 $$Q10 = \left( {\frac{{v_{2} }}{{v_{1} }}} \right)^{{\left( {\frac{10}{{T_{2} - T_{1} }}} \right)}} ,$$

where *v*_i_ represents velocity and *T*_i_ the corresponding acclimation temperature. We calculated four *Q*_10_ values corresponding to intervals of 15–20, 20–25, 25–30, and 30–35 °C. Trials were discarded if they spanned larger intervals (i.e., if an individual was only tested at 15 and 25 °C). To determine if season affected thermal sensitivity, we performed a linear mixed model with Gaussian distribution on the *Q*_10_ values. We used temperature interval and SVL as covariates; SVL was log-transformed to account for allometric effects. To account for repeated measures, we included individual ID as a random factor. All *Q*_10_ values were natural-log-transformed to meet model assumptions of homoskedasticity.

### Distribution modeling

#### Correlative niche model

We used Maxent to construct correlative niche models using native range occurrence and climate data to project onto Florida. Occurrence data for *F. pardalis* spanning from 1876 to 2022 were downloaded from various databases^62–64^ and were sorted to remove duplicate records, records without georeferences, and improbable georeferences based on locality descriptions. Only points from the native range (Madagascar) were considered; we did not include data from nearby introduced populations of *F. pardalis* on Réunion. We thinned the points at a 4 km threshold to a total of 149 remaining points.

We calibrated models using ENMeval^65^, using the ENMevaluate function with extrapolation and without clamping using default parameters and input of 19 bioclimatic layers at 1 km^2^ resolution (WorldClim2^66^) in the native range region. This method evaluates models constructed with varying combinations of regularization multipliers (0.5, 1, 2, 3, 4) and feature classes (linear, linear—quadratic, linear-quadratic-hinge, linear-quadratic-hinge-product, and linear-quadratic-hinge-product-threshold). Before running models in ENMevaluate, and to avoid potentially problematic multicollinearity in our models, we calculated the variance inflation factors (VIF) of our initial model with all 19 bioclimatic variables. If any predictor variable had a VIF > 5, we removed the variable with the lowest permutation contribution to the model. ENMevaluate was used to select the top model based on the AICc value within 2 to the lowest AIC model. We then used the top model to project onto Florida using the Maxent graphical user interface^67^ with extrapolation and no clamping and 10,000 background points.

#### Thresholded winter temperatures: cold tolerance, activity window, predicted performance

Because cold tolerance is often the most limiting factor for ectotherms at higher latitudes^68^, and because *F. pardalis CT*_gape_ exceeds typical annual maximum ambient temperatures in Florida, we chose to subset our distribution modeling to winter environmental data. To examine physiological data in the context of environmental temperatures in Florida, we created thresholds based on our thermal trait data. First, we assembled PRISM climate data for the extent of Florida for December 15 to February 15 from 2001 to 2021^69,70^ at 800 m resolution and extracted daily minimum (*T*_min_) and maximum (*T*_max_) temperatures. These dates encompass the 7 years prior to the first reported population, to account for potential lag times in reporting or discovery of this and other populations^71^. While ambient air temperature data has been criticized for use in predictive modeling frameworks (reviewed in^16^), such data may provide a decent approximation of available temperatures in this arboreal species^72^.

We used package ChillR to calculate hourly temperatures for each 800 m pixel in Florida for each day over December 15th to February 15th time period for all years. ChillR takes as input *T*_min_ and *T*_max_ as well as sunrise and sunset times, and latitude^73^. It then applies an algorithm from^74^ which models hourly temperatures by fitting a sine curve for daytime temperatures, and a logarithmic decay function for nighttime temperatures. To assess how cold tolerance (*CT*_min_) may influence *F. pardalis* distribution in Florida, we flagged the hourly temperature data where the temperature was below three different thresholds (see below) for six or more consecutive hours. The six-hour exposure time threshold accounts for time to overcome thermal inertia associated with differences in cooling rates of ambient temperature and body temperature^16^, as well as removes potential noise in the data associated with rapid but unsustained changes in temperature (e.g., a passing storm front). We counted the number of days that met this condition for each threshold for each yearly period, and then averaged the number of occurrences across those winter periods for reporting. The thresholds were: 9 °C, representing ~ 1 °C below the average measured *CT*_min_ in this study; 6 °C, representing the lowest individual *CT*_min_ recorded in this study and the lowest temperature experienced in the exclosure; and 3 °C, below temperatures experienced by chameleons in this study and below recorded *CT*_min_ for the species.

To examine the remaining thermal traits, we subset the hourly temperature data to daylight hours, as *F. pardalis* is diurnal and reliably falls asleep upon darkness (Claunch and Goodman *pers obs*). We calculated an activity window for *F. pardalis* by categorizing all temperatures during daylight hours that fell within the recorded body temperatures in the thermal preference gradient as active hours. We examined the activity window in two ways: first by averaging the number of active hours across all winters for each pixel in Florida, then by calculating the percent of active hours falling within available daylight hours across all winters for each pixel. We also calculated predicted performance during winter to serve as a proxy for the relative ability for *F. pardalis* to forage or engage in escape behaviors. To do this, we generated a performance curve using a generalized additive model of the maximum individual velocities per 25 cm segment at each test temperature bookended by velocities equaling 0 at *CT*_min_ and *CT*_max_ test temperatures, with *k* = 5 and smoothing parameter = 0.1 (Fig. 5). From this curve, we calculated the predicted performance for each daylight hour’s temperature at each pixel. We assessed performance in two ways: (1) by averaging the predicted performance across all winters per pixel; (2) by thresholding performance values that fell within 80% of maximum performance in the GAM across all winters per pixel.

#### Assessing model predictions

We assessed model predictions in two ways. First, we overlaid known established or formerly established presences in Florida on the Florida projections from all models described above, then extracted the associated data at these localities for comparison and evaluation. Because verified established presences are limited in number for *F. pardalis*, we also compared state-wide model projections produced by each metric. Second, to compare the utility of activity *versus* performance models, we normalized model outputs by setting the lowest value to 0 and the maximum value to 1; this was achieved by subtracting the lowest output value from all records, then dividing all output records by the resultant maximum value for each model. Thus, the scale reflects relative predicted activity and performance such that a value closer to 0 reflects the minimum predicted activity or performance, while a value closer to 1 reflects the maximum predicted activity or performance.

### Ethical approval

All applicable institutional and/or national guidelines for the care and use of animals were followed. All protocols were approved by USDA QA-3214 (Study Director, Bryan Kluever) and University of Florida IACUC 201,910,938. The reporting in the manuscript follows the recommendations in the ARRIVE guidelines.

### Supplementary Information

Supplementary Figures.

## Data Availability

Data for experimental determination of thermal traits are archived with USDA^75^. The data and code generated during the current study are available at GitHub [https://github.com/nmclaunch/F_pardalis_thermal].
