# Supplementary material for: Commonly collected thermal performance data can inform species distributions in a data-limited invader
Source: Sci Rep. 2023 Sep 23;13:15880. doi: 10.1038/s41598-023-43128-4 (PMC10517990; doi:10.1038/s41598-023-43128-4)
Supplement: Supplementary file 1 — Supplementary Figures. [file 41598_2023_43128_MOESM1_ESM.docx]

**SUPPLEMENTARY MATERIAL**


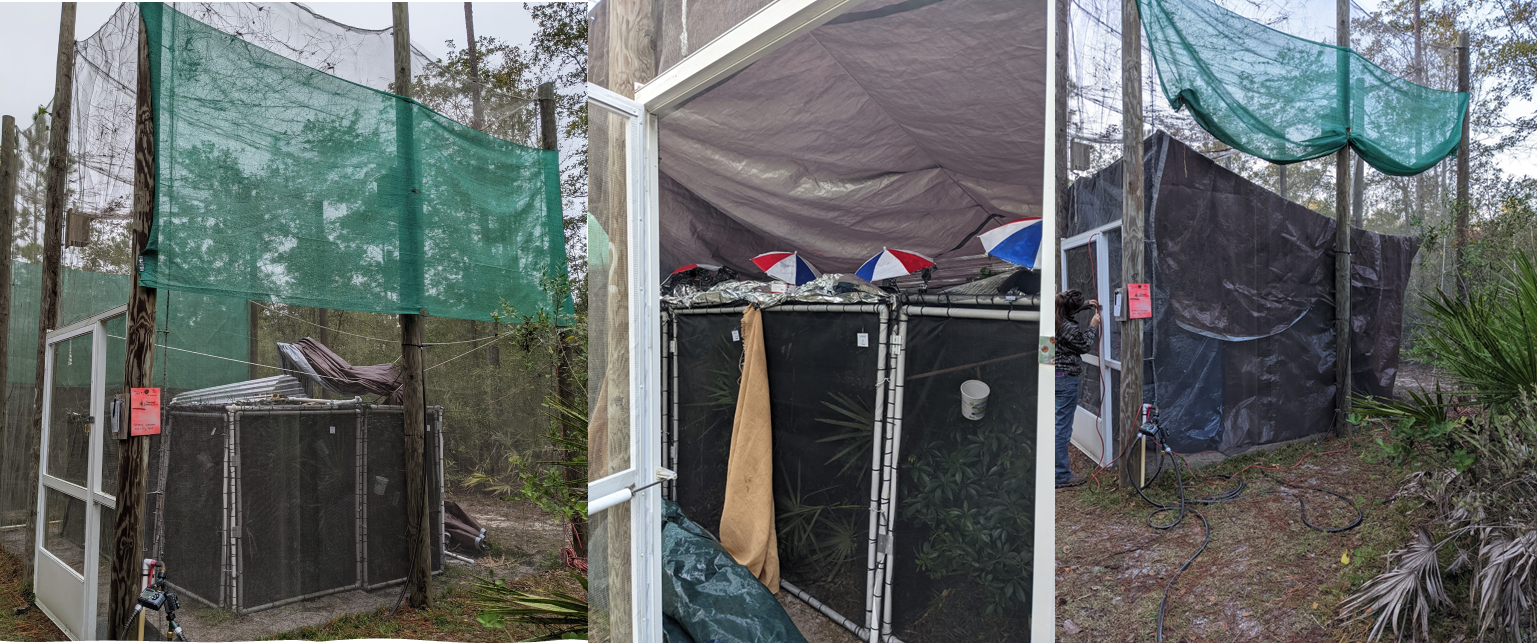


**Supplemental Figure 1.** Exclosures housing *Furcifer pardalis* at USDA Wildlife Services National Wildlife Research Center in Gainesville, Florida, from October 2020 to August 2021. Left image depicts exclosures during the majority of the study, when temperatures remained above 12 °C. Middle image depicts the inside of the set-up when temperatures fell below 12 °C, note heat emitters above individual enclosures topped with mylar-lined umbrella hats to contain heat and roof tarp covering. Right image depicts the set-up covered in tarps when temperatures fell below 12 °C. Photos by Natalie Claunch.


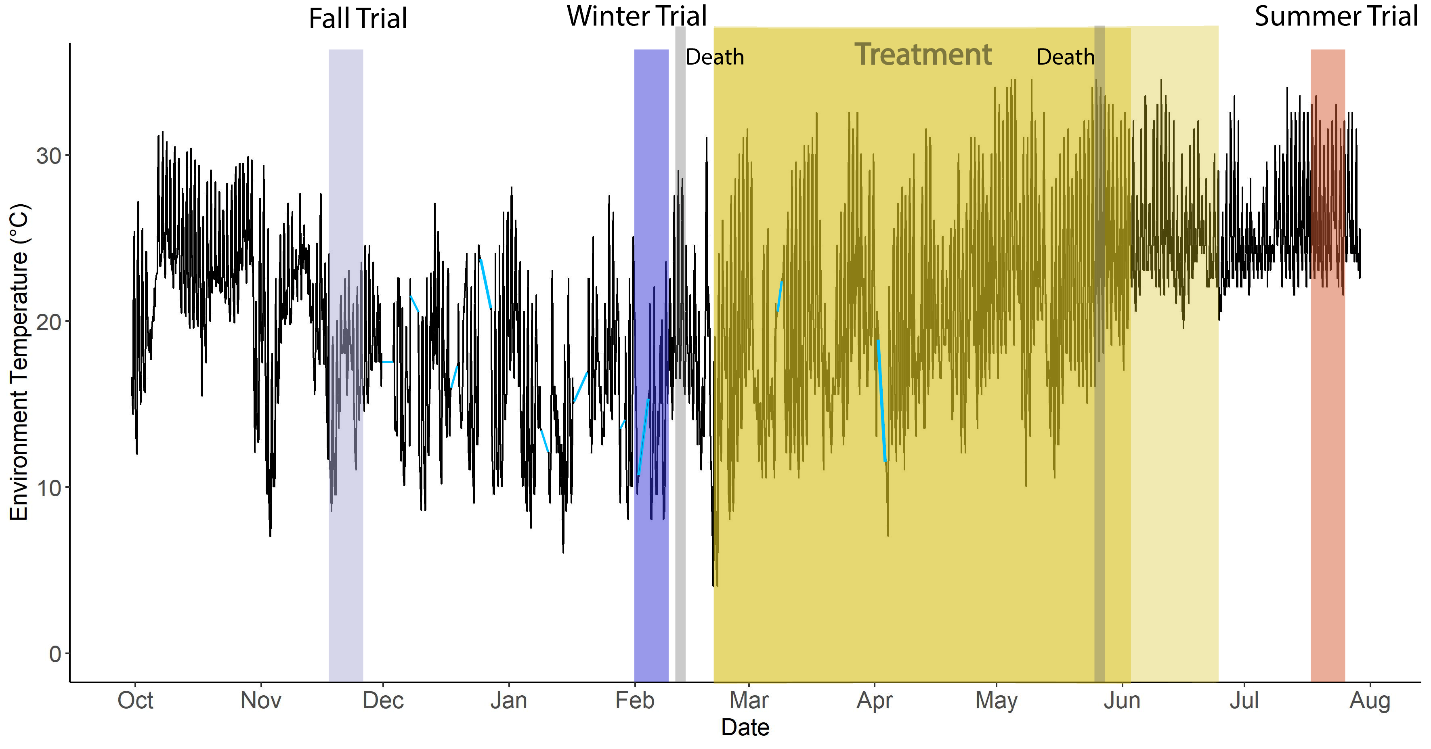


**Supplemental Figure 2.** Timeline of seasonal thermal trials (Fall, Winter, and Summer) conducted on *Furcifer pardalis* exposed to seasonal fluctuations in temperature at USDA Wildlife Services National Wildlife Research Center in Gainesville, Florida, from October 2020 to August 2021. Environmental temperatures displayed are derived from iButtons placed inside individual enclosures, and cold temperatures are within-enclosure temperatures when covered to buffer extreme cold (see Supp. Fig. 1). Line gaps, colored light blue, indicate periods when covered enclosures were not sufficient to buffer extreme cold and chameleons were temporarily housed indoors. In February, following the death of one individual (gray shading), all chameleons were treated for dermatomycosis from late February to early June (dark yellow shading). This individual was moribund and not included in winter trials. Following the death of an individual in late May, treatments were discontinued for all but two individuals until late June (light yellow shading). Spring thermal trials were not conducted due to ongoing medical treatment. Detailed accounts of disease identification, treatment, and cessation of treatment are available at Claunch et al., 2022.


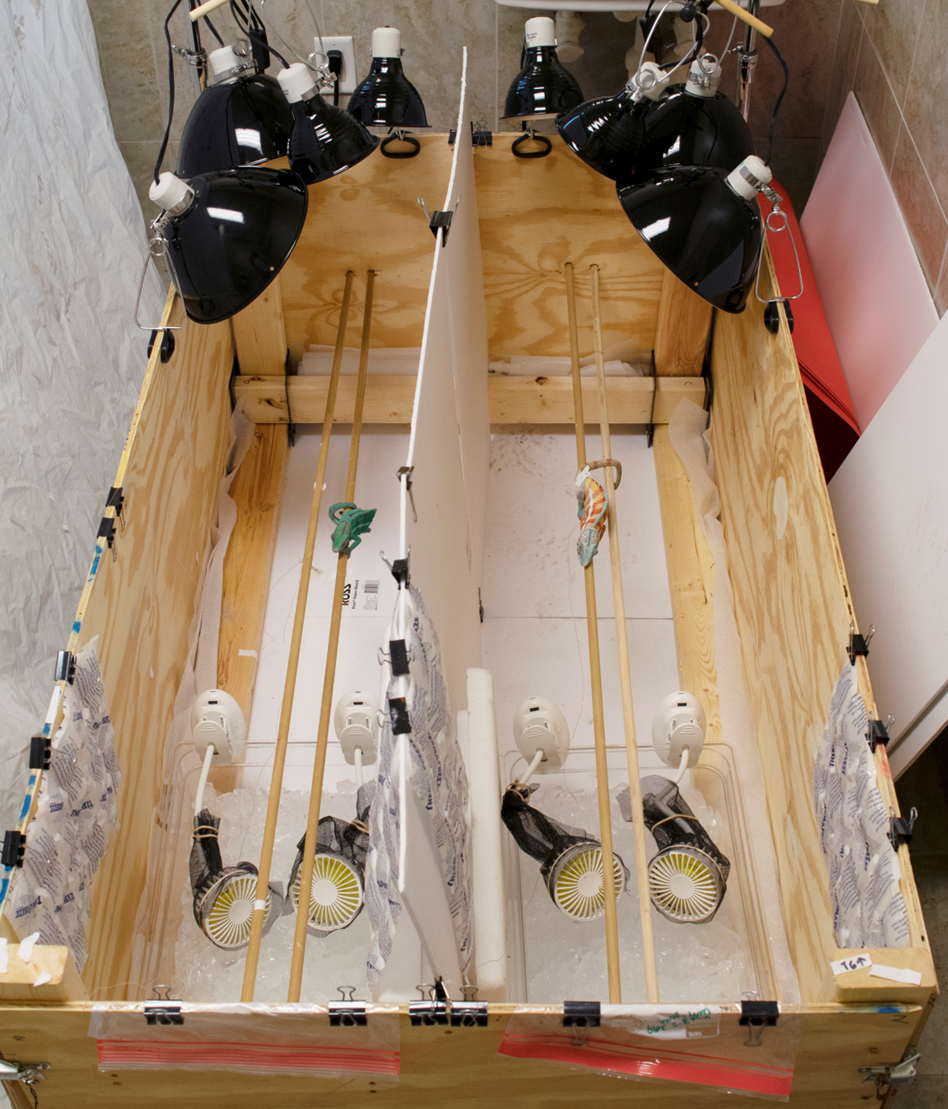


**Supplemental Figure 3.** Thermal preference arena used to assess behavioral thermoregulation of *Furcifer pardalis* after exposure to seasonal fluctuations in temperature at USDA Wildlife Services National Wildlife Research Center in Gainesville, Florida, from October 2020 to August 2021. Chameleons were outfitted with thermocouples with long leads that allowed them to explore the arena unobstructed and a curtain drawn to reduce bias caused by reaction to human observers. Photo by Natalie Claunch.

**
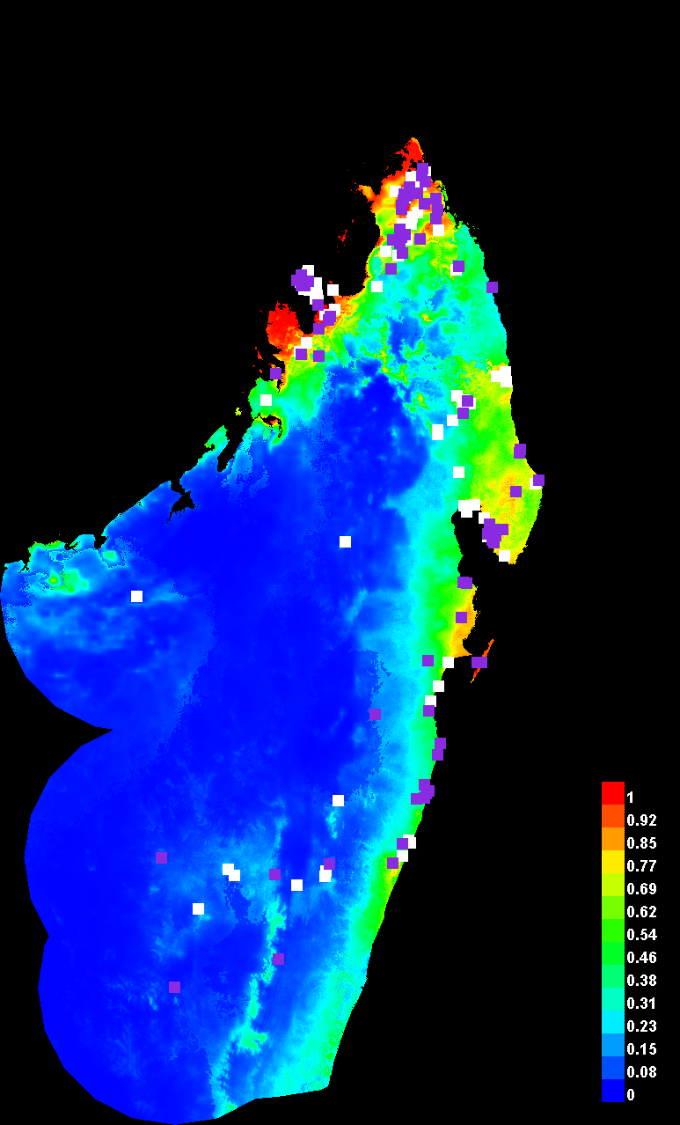
**

**Supplemental Figure 4.** Predicted suitable area for *Furcifer pardalis* in its native range of Madagascar from the top correlative model based on occurrence data using MaxEnt. Warmer colors indicate area predicted to be more suitable. White squares indicate presence points used for training, while purple squares indicate presence points used for testing.


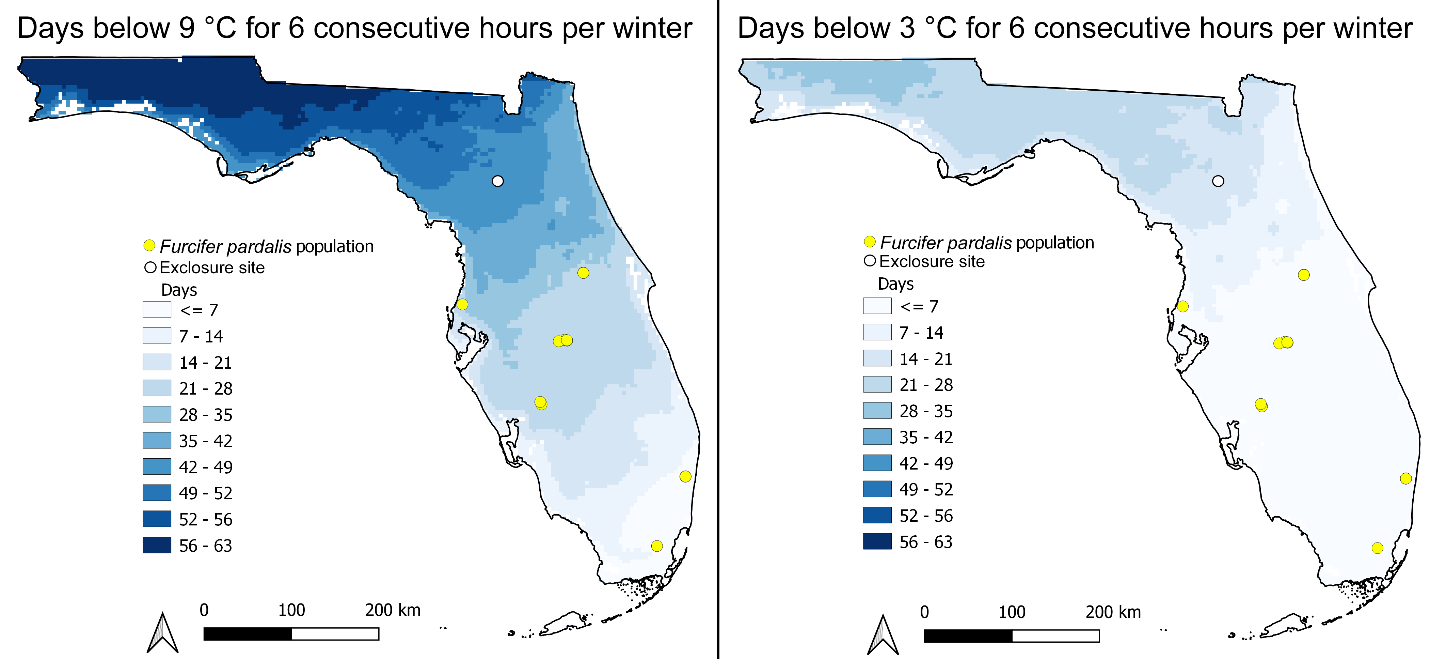


**Supplemental Figure 5.** Average number of days during each winter period (December 15- February 15) in Florida, USA, where temperature fell below the 9 °C (the average critical thermal minimum of *Furcifer pardalis*), left, and 3 °C (below the lowest measured critical minimum for *F. pardalis*), right, for 6 or more consecutive hours, from 2001-2021. Yellow points indicate locations where *F. pardalis* populations have established during this period. White point indicates the location of the experimental exclosure at USDA Wildlife Services National Wildlife Research Center in Gainesville, Florida.


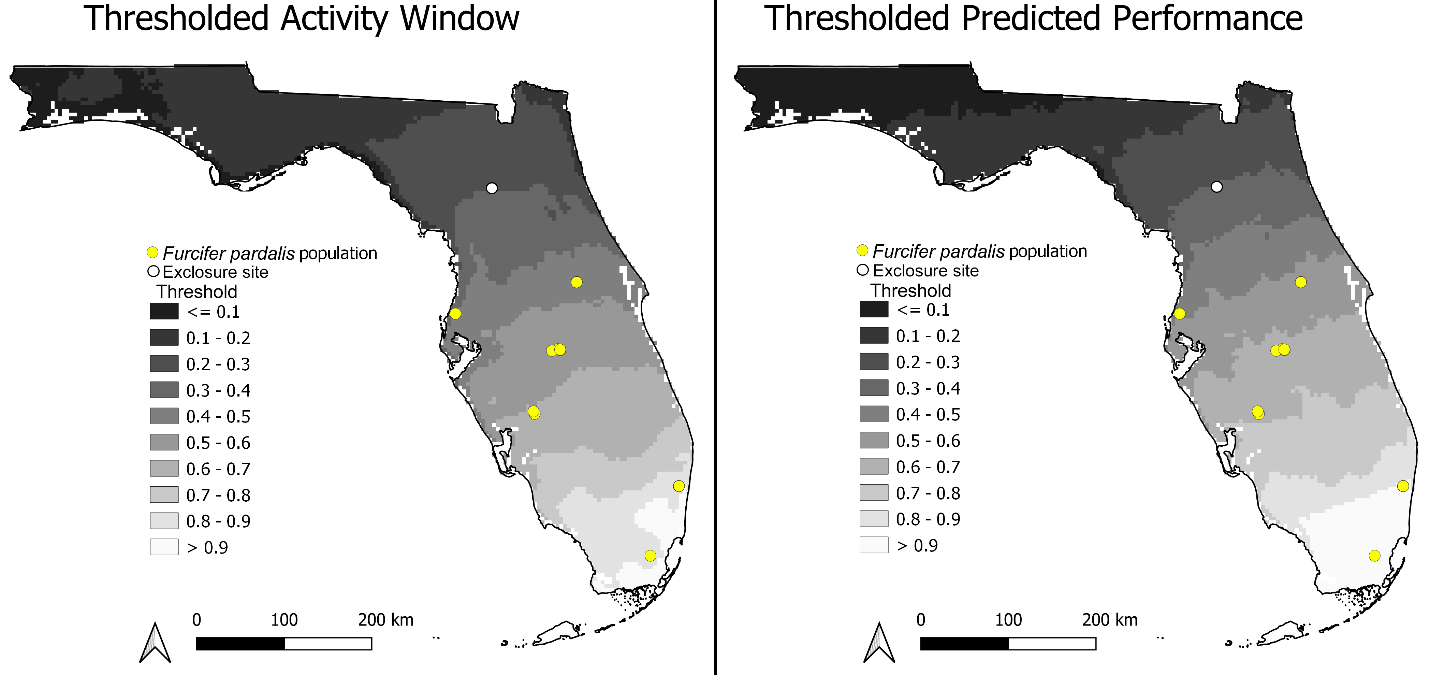


**Supplemental Figure 6.** A comparison of model outputs for predicted activity window and predicted performance of *Furcifer pardalis* for winter temperatures (December 15- February 15) from 2001 to 2021. Outputs were normalized to a 0 to 1 scale by subtracting the lowest value from all areas, then dividing all areas by the highest value. While predictions from each output are not drastically different, the activity window, calculated based on thermal preference data, is more conservative than output from performance data. Yellow points indicate locations where *F. pardalis* populations have established during this period. White point indicates the location of the experimental exclosure at USDA Wildlife Services National Wildlife Research Center in Gainesville, Florida.
